# Is there a difference in the perception of outpatient clinic teaching, between medical students and teachers'? A mixed method study

**DOI:** 10.15694/mep.2019.000080.1

**Published:** 2019-04-12

**Authors:** Bander Dallol, Birgit Fruhstorfer

**Affiliations:** 1 University Hospital Coventry and Warwickshire; 2 Warwick Medical School

**Keywords:** Teacher's perception, medical student's perception, outpatient clinics teaching, undergraduate teaching, mixed method project

## Abstract

This article was migrated. The article was marked as recommended.

**Background:** Outpatient clinic teachingis an important part of undergraduate medical student’s experience. Increasing pressure from service commitments has meant that limitations to the learning process have continued to surface. Previous studies have looked at satisfaction level of all involved; however no studies have examined whether the limitations are perceived differently between students and teachers in a real life setting.

**Method:** A mixed method project including on-line questionnaires, focus group and interviews with ninety two participants. This was conducted in a local university hospital. Teachers and students were asked about teaching styles undertaken and limitations during clinics. They were also asked about how to improve the learning process during outpatient clinics.

**Results:** Teachers and students agree that seeing patients under supervision is the ideal teaching style during clinics. Both groups agree that time and space, are the obvious limitations to outpatient teaching. Advanced planning, however, and teachers’ attitude toward teaching have also been rated highly from students’ perspective.

**Conclusion:** Common themes emerged between students and teachers regarding outpatient teaching. Reducing patient numbers seems the ideal solution. Given the increasing service demands in the current climate, it was perceived that improving communication before and after clinics between students and teachers who are interested in teaching could positively influence learning in this setting.

## Introduction

Teaching medical students can take various forms. While lectures and seminars provide students with theoretical knowledge, bedside and outpatient clinic teaching help to apply this knowledge into real life practical experience. Outpatient clinic teaching has an important role in undergraduate education (Almoallim et al, 2015; Wood, 2003). There is also evidence that this practical aspect of participation is a more effective way of learning ( Schmidt, 1993).

While ward-based teaching can provide a reasonable platform for practical experience, it can be compromised by the limited number of patients and the nature of conditions presented ( Dent, 2013; Denton et al, 2010). The General Medical Council (GMC) suggested that greater use is made of outpatient clinics for students teaching in order to combine scientific knowledge with practical experience (GMC, 1993 & 2009).

There are many theories as to why outpatient settings provide a suitable and even superior environment for undergraduate education. Lawson & Moss (1993) suggested that undergraduate teaching has shifted from inpatient to outpatient mainly because of reduced number of days inpatients spend in hospitals and that there was increase of ambulatory patient contact by 70%. Personality of the teacher and the environment where students are taught does affect the learning style for undergraduate medical students as explained by Ramsey et al (1988)

Despite all this,outpatient clinic is still one of the most underused resources of teaching ( Eaton & Cottrell, 1998). Various studies and papers have looked at effectiveness of learning in outpatient settings (Butterfield et al, 1991; Kerfoot, 2002; Davis & Dent 1994; Neumayer et al, 1998; and Wood, 2003). Some looked at students satisfaction ( Hajioff & Birchall, 1999) while others examined and different styles and suggested ways in order to aid students learning ( Dent, 2013; Denton 2013).

Considering the clear role of outpatient teaching on undergraduate education, there are still long-term restrictions to the learning process. This includes time and space. Quality of teaching is also dependent on the teacher’s willingness and attitudes towards teaching in outpatient setting ( McGee & Irby, 1997).

This project will aim to explore whether there are differences in the perception between teachers and students. The project will outline any differences in teaching styles undertaken during clinics, limitations or barriers to outpatient teaching, the level of enjoyment during outpatient clinic teaching and finally ask those involved for suggestions on how to improve the quality of teaching in outpatient clinics, at a local trust.

## Methods

### Study design

**Figure 1.**
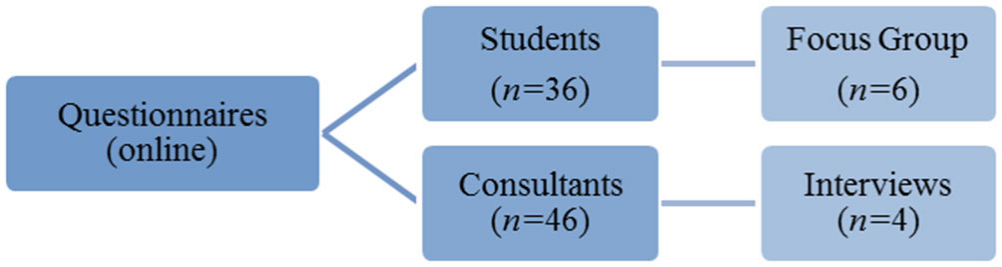
Study design

This is a mixed methods study, using questionnaires, a focus group and individual interviews ( Figure 1). A parallel convergent design was adopted in this study ( Creswell & Clark, 2011) this study design was chosen as data were collected and analysed concurrently in the same phase.

The on-line questionnaire was completed, using Qualtrics software. Most questions were closed questions with multiple choice answers and Likert scales. Participants were asked about teaching styles, limitations and enjoyment of outpatient clinics. There was one open question where participants were asked for their comments on how to improve outpatient teaching. The questions were slightly different for each group. (Supplementary File 1 & 2)

The qualitative part consisted of one focus group for medical students and four individual consultants’ interviews. Questionnaires, interviews and focus group questions were piloted beforehand with a medical student and a consultant to ensure relevance and ease of completion (Supplementary File 3). Those who were involved in the pilot phase did not participate in the meetings.

### Setting and Participants

The course at Warwick Medical School is designed for graduate students. This means that all students must have undertaken a relevant undergraduate degree before enrolling into the course. The course is for four years. Year one students teaching is largely delivered at campus, therefore they are not expected to attend outpatient clinics. The majority of learning from year two is based in the community and partners trusts. This includes three local hospital trusts and GP practices. Students are encouraged to attend outpatient clinics during this period (Warwick Medical School, 2018).

Invitation to second, third and fourth year medical students were sent via Warwick Medical School Head of Education. All consultants at a local teaching hospital trust were invited to participate in the study. Permission was sought from all clinical directors (Supplementary File 4). Consultants then were emailed directly by the main author (Supplementary File 5).

Both groups received Participant Information Leaflet alongside the invitation email (Supplementary File 6). They were also given the opportunity to participate in the qualitative part of the study by directly emailing the main author. A prize draw was offered for participation. An email reminder was sent before closing date.

Although the main author is employed by one of the local trusts, Student who took part in the qualitative part of the study had not been in placement with the main author and consultant interviewees were not based in the same department.

### Data collection

#### Questionnaires

On-line questionnaires data were collected during April and May 2018 from both groups. The closing date was set in May after which the surveys were not accessible. Students attending other partner trusts were included in the study. Consultants on the other hand were all from one trust only. Consent of participants was assumed by completion of the questionnaire. Closing date for completion was in May 2018 for both groups. There were 36 students and 46 consultants responded.

#### Students’ focus group

Six students participated in the focus group, one female and five males. Two were in their third year and four in their second year of training. The meeting took place in April 2018. All participants signed a consent form at the start of the meeting (Supplementary File 7). The meeting lasted for an hour as data saturation was reached at that stage. Students were asked a series of similar questions to online questionnaires (Supplementary File 8). All participants were included in the discussion. Discussion was mainly informal and field notes were taken to aid transcription and theme setting. The focus group was arranged at a time and a place convenient for participants. The focus group schedule was also prepared beforehand and used to guide the meeting (Supplementary File 9).

#### Consultants’ interviews

Four consultants’ interviews took place during April and May 2018. All interviews were conducted at a local hospital. Consultants were from Neurology, Medicine, Surgery and Ophthalmology departments. The average length per interview was 30 minutes. All interviews were recorded and field notes were taken during the interviews to aid transcription and theme setting. Data saturation was reached after answering all interview questions (Supplementary File 10).

### Data analysis

#### Questionnaires

Questionnaire data were analysed via Qualtrics software. Descriptive analysis was mainly used due to the nature of data gathered ( Creswell & Clark, 2011). Content analysis was conducted for the open questions and was summarised in the results section.

#### Focus group and Interviews

All focus group and interviews data were transcribed and coded into themes, by the main author, using NVivo software. All data were anonymised during transcription to ensure confidentiality.

#### Ethics

Biomedical and Scientific Research Committee (BSREC) Course-Delegated Ethical approval and a Governance arrangement for Ethics Committee (GafREC) approval were granted, by Warwick University and the local trust Ethics Committees respectively.

## Results/Analysis

**Figure 2.**
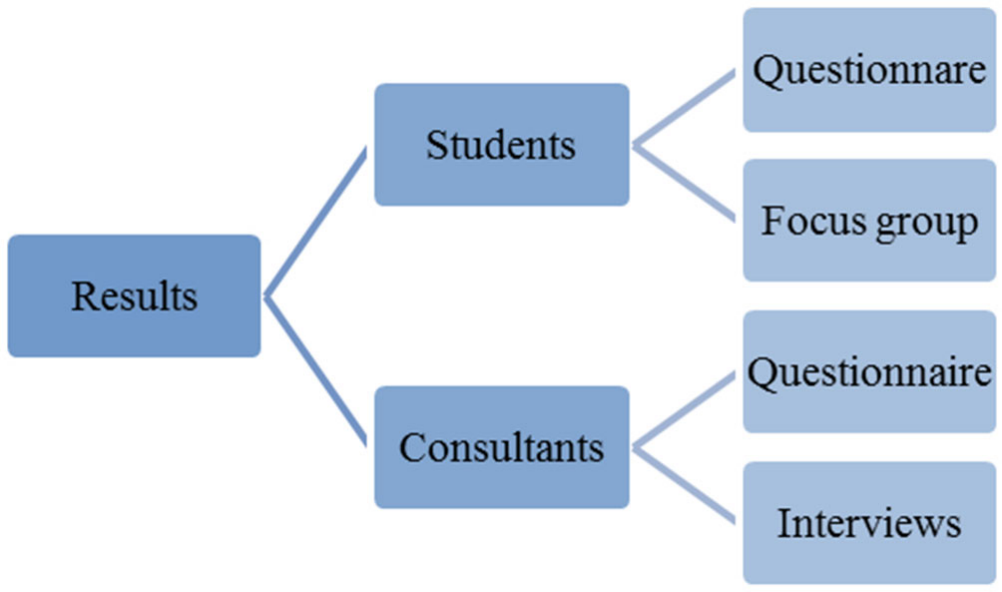
Results analysis model

This was analysed and presented as in Figure 2:

### Students’ results

#### Questionnaire

Online questionnaires were completed and returned by 6% (36/600) of students. Response rate was comparable between the three year groups, 13, 12 and 11 responders, respectively. The majority of clinics were attended at one hospital trust (61%) and 32% were at two other local hospital trusts, 16% at each. The remaining 7% were at local GP practices.

Most students ( *n=*30) were in their twenties. Four were aged 30 to 40, and two were from >40 age group. There were more female than male respondents (61 vs 33%). Most students in this survey attended medical clinics (80%), 16% attended a surgical and the rest attended other clinics. Students participated in the study attended a mean average 24.77 clinics per year (SD 13.22).

Students were asked about the most preferred teaching style during outpatient clinics. Most respondents preferred to see patients under observation, see table 1:

**Table 1.** First preference of teaching style selected by students

Teaching style	% of students (number of students)
See patients under observation	55% ( *n=*20)
See patients independently	22% ( *n=*8)
Discuss with teacher in-between patients	16% ( *n=*6)
Observe	5.5% ( *n=*2)
Discuss at the end of a clinic	0

**Figure 3.**
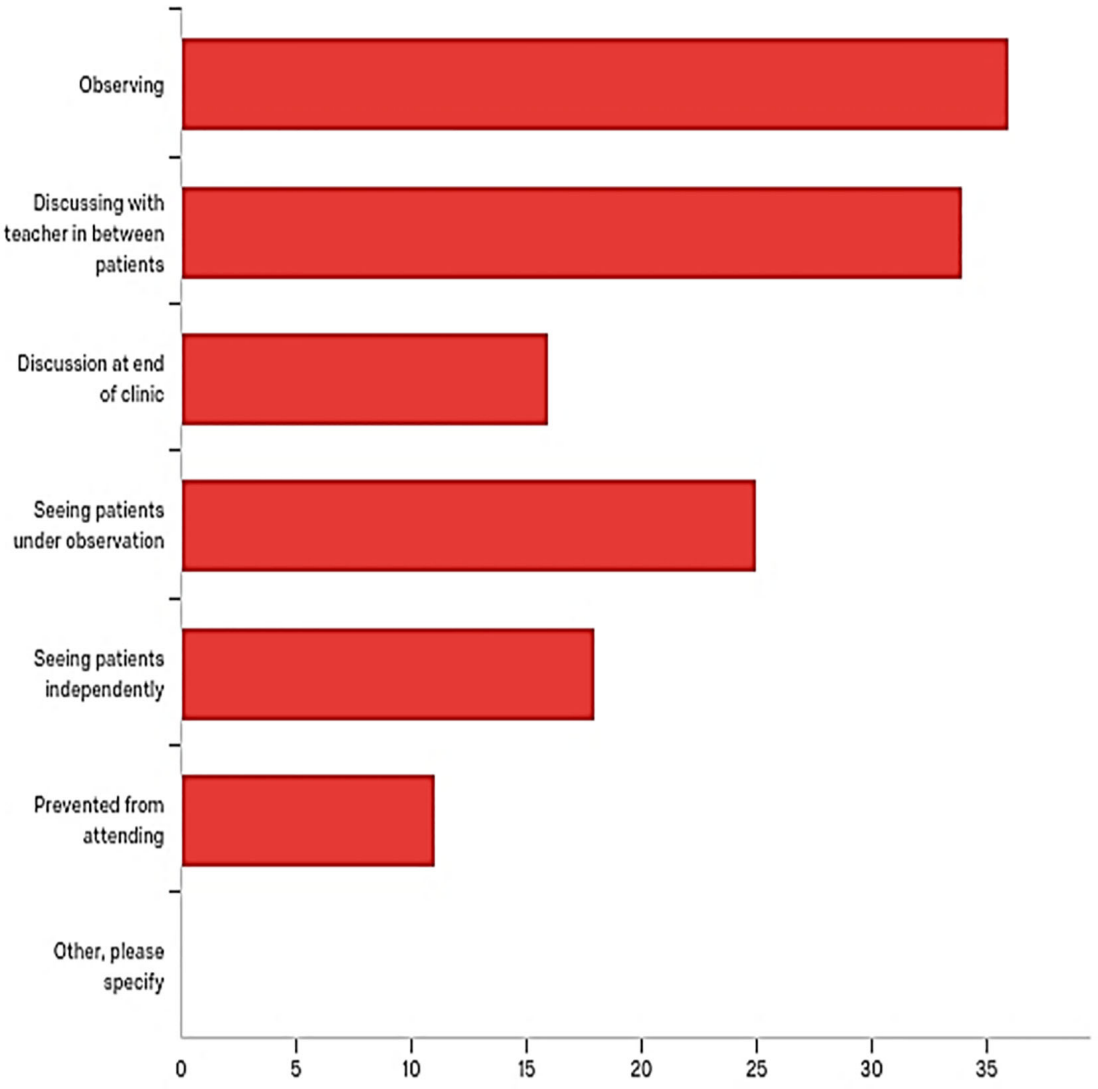
Number of students who have experienced different styles of teaching during the clinics they have attended

Respondents were asked about the different styles of teaching adopted during clinics they have attended. Observing during clinic, followed by discussing between patients and seeing patients under observation were the most experienced learning styles ( Figure 3). Student’s response did not differed between trusts.

**Figure 4.**
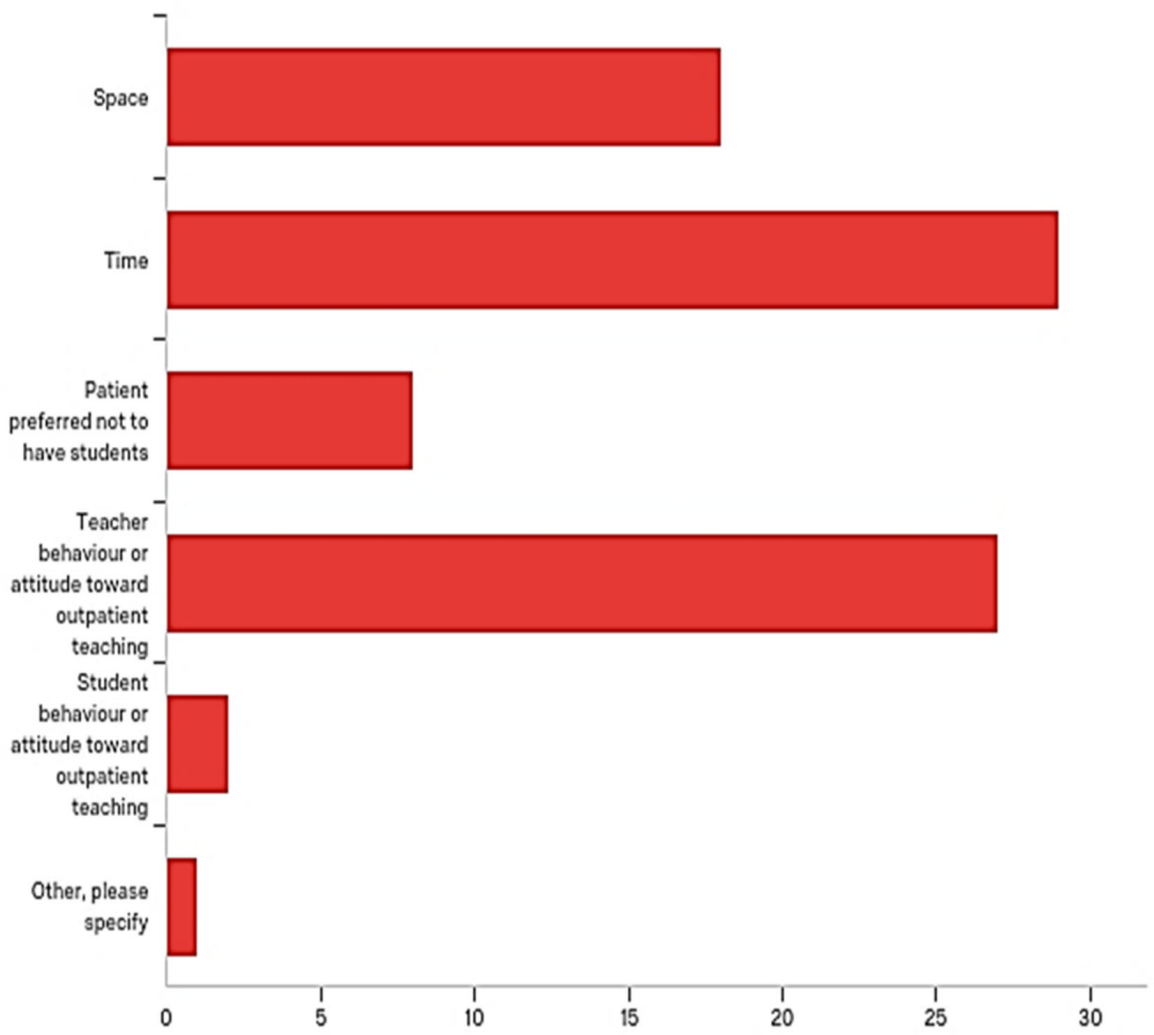
The number of students selecting each limitation to outpatients teaching

Most respondents indicated that, time was the main limitation to outpatient teaching, followed by teacher’s interest or attitude toward teaching. Space was the third on the list of limitations ( Figure 4).

Respondents indicated, on scale of 1-10 (where 10 is very strongly agree), that they feel outpatient teaching has a positive influence on learning, mean value of 7.39 (SD 1.65). The majority (80%) of students, who completed the questionnaire, ‘enjoy’ or ‘very much enjoy’ learning in outpatient clinic in a five points Likert scale (Appendix 1).

In the open question the students were asked; how to improve outpatient teaching. Responses were themed and accompanied by example quotations in table 2 below:

**Table 2.** Main themes from students’ comments on how to improve outpatient teaching and number of students (N) commented on each theme

Theme	N
**To see patients under observation/ independently:** *‘Medical students to see at least one patient independently during clinic’* **Teachers attitude to teach:** *‘Having an enthusiastic teacher. This is the single biggest factor affecting utility of clinics for me’* **Discuss during clinics:** *‘Generally good learning can happen when the doctor (teacher) involves the student in some aspects of the consultation...’* **Teaching clinics:** *‘Having specific teaching clinics which students can sign up for will help the learning process...’* **Time and space:** *’ More time and space to be able to do student consultations under observation or independently in a separate room and then report back’*	1210864

#### Focus Group

The Data from the focus group was analysed via NVivo software and coded into themes. This has revealed three main themes; teaching style during clinics, limitations to outpatient teaching with three subthemes, and how to improve teaching.

*Teaching style.* Participants reported different styles during clinics and this includes; observing, discussing between patients, active participation, fully or partially, during history taking or examination or both. The style also seemed to differ depending on the type of clinic and the student’s year of study.

..You are either sat in the corner quietly throughout or like a piece of furniture in the room that is listening or you are questioned in between patients very intensively..

Focus group participants preferred a mix of styles depending on clinic type, year of study and confidence of the student. In orthopaedics clinics, for instance, the most preferred style during outpatient teaching is seeing patients under observation. While in medical clinics discussing between patients initially and then seeing patients separately was more desired.

I think personally I prefer having a bit of a mixture of being involved and also having discussion and having time to ask questions or answer questions, a bit of a combination

*Limitation to outpatient teaching.* The most common limiting factor to outpatient teaching from participants’ point of view was poor communication and preparation before clinics. All six participants motioned this to be an important limitation. This included information given by the university about clinics available, or with individual department and the consultants they are assigned to. This can results in clash between students seeking the same clinic or between students and other disciplines, like nurses, physician associates and junior doctors.

I think one of the limitations is simply on probably availability I would say, on being able to, or knowing what clinics are going on even. Because again, unless you happen to know about things because you know of a certain consultant or the consultant you are assigned to that sort of thing if you have nothing to go on it is a bit more hit and miss.

Teachers’ interest and attitude was also highly featured in participants’ responses. Five students considered teachers’ interest and willingness to involve them during consultation is vital to the success of learning process.

Being willing to get students involved, even to a small extent from asking a few questions during the consultation about the disease area to getting students to do parts of the history or exam, or interpret an investigation.

Another student suggested that wrong technique or rushed examination by the teacher can negatively influence student’s behaviour.

‘We can pick up bad habits by watching the quick examinations’

Time was the third most common limitation featured. This was mentioned by four participants to be one of the limitations to outpatient teaching.

*How to improve teaching.* Students in this group agree that preparation and prior agreement with consultant is the most important step in order to improve not only students’ attendance but also the learning experience during clinics. This will allow students to prepare before clinics and insure no clashes with other students. There were few other suggestions which are summarised with example quotation in table 3 below:

**Table 3.** Focus group participants’ suggestions on how to improve outpatient teaching

Information about clinic times and whereabouts: *‘giving forms out when you are on a certain block, these are all the different clinics going on, so that you can organise your own learning’* Understanding medical school structure: *‘Consultants should read a quick A4 document from the medical school that says in 1st year they are doing this, in 2nd year, 3rd year ,4th year this...’* Preparation before clinic: *‘...read up on the subject that I will be doing so I had proper questions to ask and refreshed my knowledge’* Engage students during clinic: *‘while you are doing your notes or dictation say read through that and tell me about the next patient’* Involve other trainees in teaching: *‘Registrars are an unutilised resource and a lot of them are interested in teaching...’*

### Consultants’ results

#### Questionnaire

Out of 410 consultants, 46 (11.2%) completed the on-line questionnaire. The mean number of years spent at consultant level was 9.35 (SD 6.72). Most consultants (95%) did two to three clinics per week, mean number of 2.59 (SD 1.03). Four percent of consultants do not have any outpatient clinics commitments as part of their role. Consultants had about two students sat with them in clinic, mean number of 1.52 (SD 1.47).

There were similar numbers of male and females respondents ( *n=* 24 vs 21). All respondents were at a consultant level amongst which 6.5% (3/46) were professors or associate professor. Respondents were from a wide range of specialties.

**Figure 5.**
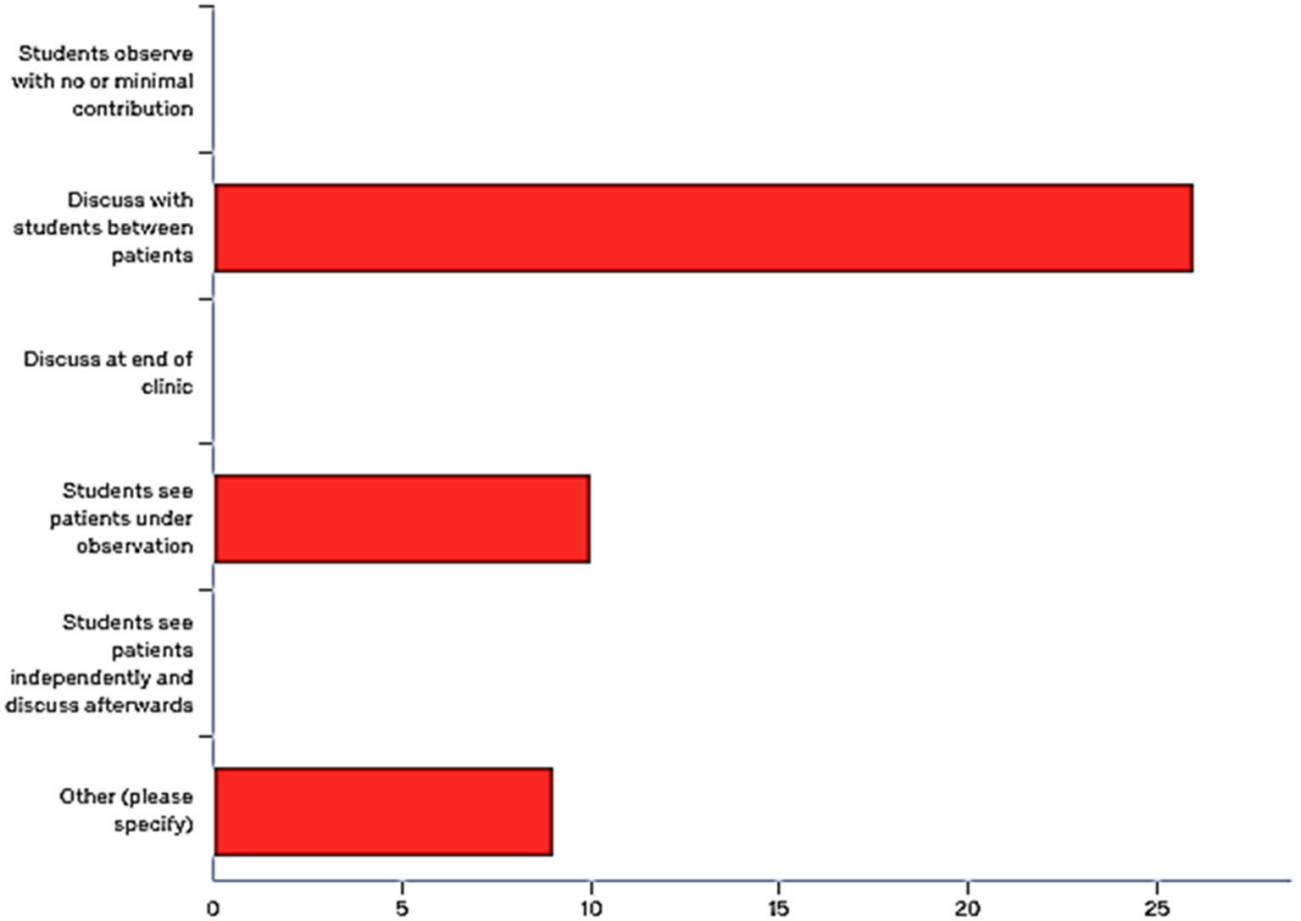
Consultant’s teaching style during outpatient clinics

Consultants were asked to choose their usual adopted teaching style during outpatient clinics, more than half ( *n=*26, 57.9%) indicated that they discuss with students between patients. 22% ( *n=*10) chose ‘see patient under-observation’ and 20% ( *n=*9) used a combination of methods or had no students allocated to them. None of the respondents chose the other three styles listed in the questionnaire ( Figure 5).

**Figure 6.**
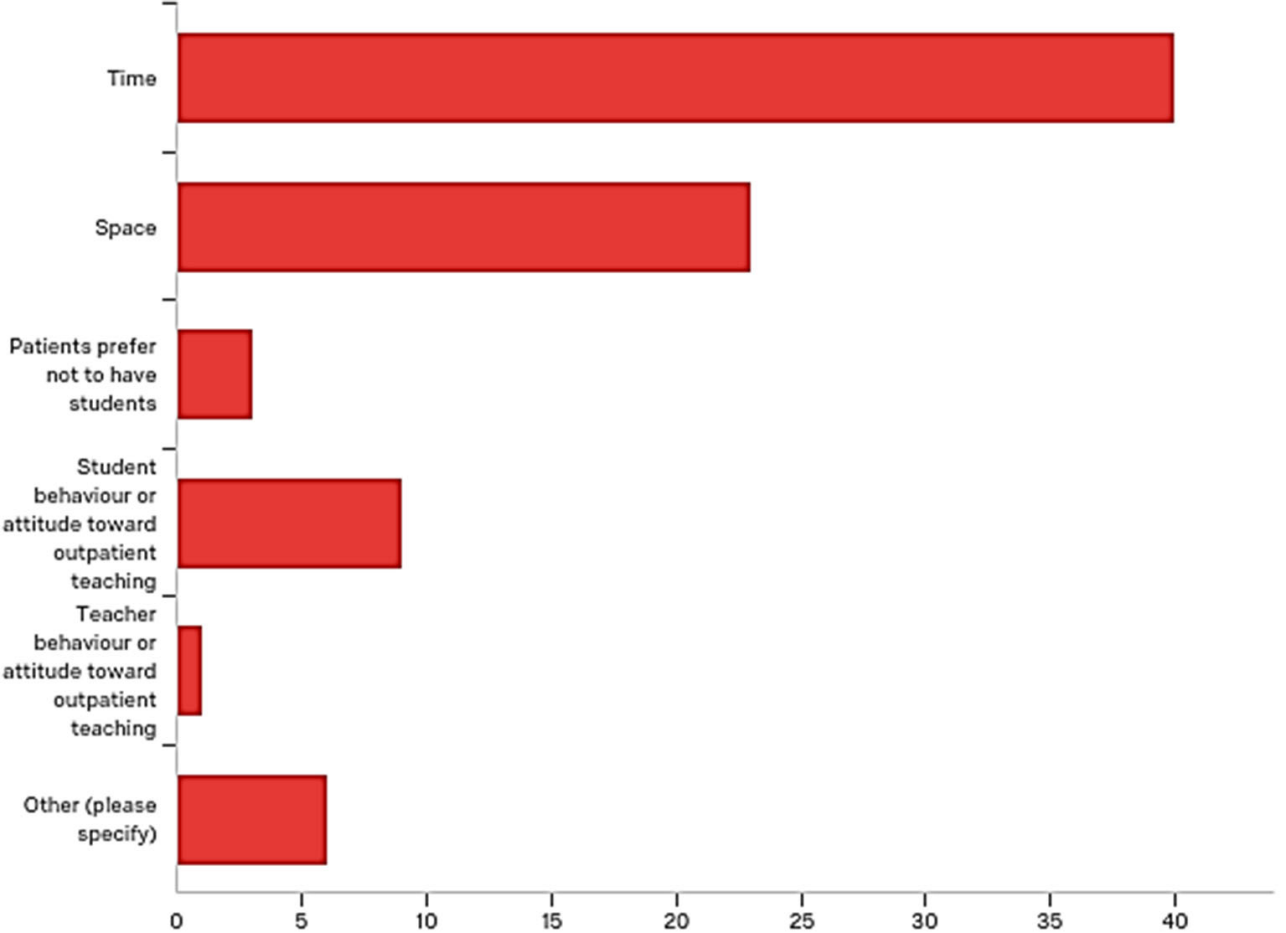
Most chosen limitations to outpatient teaching by consultants

Teachers were then asked about the limitations, they face, to outpatients teaching. Time and space were the most chosen limitations by consultants 40/46 ( Figure 6):

Six consultants who completed the questionnaire did not answer the question about their preferred teaching environment. Of those who answered, more than half (57%, 23/40) chose a combination of outpatient and inpatients to be their preferred environment for teaching.

Consultants were asked to indicate how much they enjoy outpatient teaching on a scale where 5 is ‘very much enjoy’, the mean score was 4.1 (SD 1.58).Respondents were then asked for their suggestions on how to improve outpatient teaching. Their answers were themed, in table 4 below, alongside example quotations.

**Table 4.** Main themes from consultants’ comments on how to improve outpatient teaching and number of consultants (N) commented on each theme

Theme	N
**Time and space:** *‘Time for student preparation and briefing before and after consultations/ separate space for students to examine patients’* **Teaching clinics:** *‘Separation of teaching and service clinics so that those that wish teach and have the skill to do so have the time to give to students.* **Advanced notice/planning:** *‘Prior warning that students will attend. Better preparation of students by medical school and student self-preparation.*	18157

Quarter of the respondents had specific training in outpatient teaching (12/46). Of those who didn’t have any training, 62% (23/37) would consider having such training.

#### Interviews

Four consultants volunteered to take part. Only three interviews were analysed as the fourth interview was not recorded due to equipment failure, however, field notes were taken during all interviews. Consultant’s experience at this level was ranging between 5 and 30 years. There was one female and three males. After analysis, four themes were identified; teaching styles, limitations, how to improve outpatient teaching, and their views on teach the teacher courses.

*Teaching styles.* Teaching style greatly varied between consultants, the type of clinic and the complexity of the patient. In this group of consultants, teaching style practiced mostly is not the preferred style for that consultant, however due to limitations (as outlined below) their teaching style has changed to adapt to circumstances. Consultants seem to employ a mixture of teaching styles depending on multiple variables, like number of patients and or students, complexity of patients and room availability.

*Limitations to outpatient teaching.* The most common and recurring limitation is time, which was cited by all four consultants in this group, followed by space which was mentioned by three of them. Student interest and lack of preparation has also featured by one of the consultants.

I find it incredibly frustrating when I am getting by week 6 and they still can’t tell me what the common drugs for [a condition] that shows me a lack of interest and a lack of engagement in wanting to make the most of that opportunity

*How to improve outpatient teaching.* Provide more time and space where students can see patients independently. Preparation and meeting with the consultant beforehand to set goals was vital for a successful learning opportunity. The ideal way to improve teaching process in one consultant interview was to ask for feedback.

Their feedback will be extremely valuable. Before you start teaching there should be a named objective, with the medical students, what we are going to learn.

*Teach the teacher courses.* Three out of fourConsultants in this group did not think teaching courses would be beneficial. One even suggested that unless consultants have interest in teaching, teaching courses will not be helpful.

‘You might teach them how but will they ever even apply it, because they don’t have the interest? Whereas those of us that have the interest probably also have the natural ability to do it as well. So it may or may not make a difference’

## Discussion

The most preferable teaching style from student perspective was seeing patients under observation, followed by seeing patient independently. This matched the finding of previous studies ( Croft et al, 2012; Hajioff, Birchall, 1999). The least preferred teaching style during clinic, from students’ perspective, was ‘observing’. This however was rated by students as the most practiced style. Teachers indicated that ‘discussing between patients’ was the most practiced. This difference in results could be influenced by responder bias. Consultants with an interest in teaching might have been more likely to participate in the study. Their teaching style may be different to those who did not respond. Students on the other hand may have been more likely to respond if they were dissatisfied with the system and saw this as an opportunity to share their experience and views. From the qualitative data it was clear that a mix of style was employed during outpatient teaching reported in both groups.

Both students and teachers enjoy outpatient clinic as a teaching environment. There were more teachers than students scoring high on the enjoyment scale. This again might be influenced by the fact that teachers interested in teaching are more likely to take part in this study. Most students in the survey attended clinics where they would only observe. which can explain the lower score in satisfaction compared to teachers. The level of student satisfaction was increased when given the opportunity to see patients independently ( Azher, et al, 2013).

Creating teaching clinics were strongly advocated, by teachers and students alike, in order to eliminate time and space constraints. This was also recommended by Dent (2005) and Latta et al (2013). However, in the current climate of increasing service pressures, it is understandable that this cannot be easily achieved. In this study, from students’ perspective, some environmental variables like time and space can have less impact than teachers’ attitude towards teaching and their willingness to allow active participation during clinic. Involving students, to some extent, during outpatient clinics has been shown to be one of the most important influential factors on the learning experience (Hajioff, 1998; Irby, 1991; McGee & Irby, 1997).

Both groups agree that prior planning and preparation before clinics, as well as having time for feedback, can make outpatient learning more effective. Setting objectives with the student and understanding the course structure is vital from students’ point of view, in order to deliver teaching at the right level. This was also recommended by Eaton & Cottrell (1998) and Dent et al (2009).

Most consultants in this study are happy to consider specific training in outpatient clinic teaching. In fact, some royal colleges are suggesting that only consultants who have undergone formal teaching training should have students placed with them ( Eaton & Cottrell, 1998). It is not the content of the teaching itself that is important here, it’s rather the planning before clinics and the role of reflection ( Anderson et al, 1997).

### Strengths and Limitations

The mixed method design provided a broader and more comprehensive understanding of the issues around outpatient teaching than either qualitative or quantitative would have explored individually. Lastly, this study is the first study, to our knowledge, looking at the difference in perception between students and teachers, of outpatient clinic teaching.

There was low response rate from both groups to the on-line questionnaires. Response rate from consultant group was higher compared to students (11.2% versus 6%). One reason for this, that could be stipulated, is some students received the invitation after their exams. It is also hard to generalise the results as consultants were recruited from only one trust. Despite this, the findings from this study were supported by finding from other studies.

## Conclusion

The study found that both groups have similar perception to the limitations of outpatient teaching. This includes time and space, preferred teaching style and on how we can improve outpatient teaching experience. Suggestions mainly focused on advanced planning and communication before and after clinics as well creation of teaching clinics. Teachers’ interest was highly regarded to be one of the main factors affecting the learning experience. Understanding course structure can aid delivery of teaching at the right level. Both groups also enjoy outpatient teaching and regard it to be highly beneficial when conducted correctly. Future studies could explore further how limitations can be resolved if teaching clinics could not be achieved at a national or regional level.

## Take Home Messages

• Trusts are encouraged to invest in teaching clinics idea where feasible
• Teachers are encouraged to engage with medical students during clinic time in order to improve the learning experience
• Students are encouraged to give prior notice for teachers before attending clinics and to read about subject matter beforehand

## Notes On Contributors

Dr B Dallol MRCP MMedEd. Masters degree student at Warwick University and a Consultant in Stroke Medicine at University Hospital Coventry and Warwickshire.

Dr B Fruhstorfer FRCS DOHNS MMedEd MPH. Project supervisor at Warwick University.

## Appendices

### APPENDIX 1

Students’ level of enjoyment of outpatient clinic learning (level 1-5, where 5 is the highest, against number of students)
