# Supplementary material for: Is there a difference in the perception of outpatient clinic teaching, between medical students and teachers'? A mixed method study
Source: MedEdPublish (2016). 2019 Apr 12;8:80. [Version 1] doi: 10.15694/mep.2019.000080.1 (PMC10712604; doi:10.15694/mep.2019.000080.1)
Supplement: Peer review history

# Reviewer report: Reviewer response for version 1

**DOI:** 10.21956/mep.19810.r29824

**Published:** 2019-11-16

**Authors:** Sateesh Babu Arja

**Affiliations:** 1 Avalon University School of Medicine

This review has been migrated. The reviewer awarded 4 stars out of 5 I thoroughly enjoyed reading this paper. This is an important manuscript on outpatient teaching, which is going to play a much more important role in the future as inpatient exposure is becoming difficult, especially in rotations like Pediatrics and Family Medicine for undergraduate medical school. I am from a Caribbean background where students do clinical rotations in the USA. These days Caribbean students getting inpatient exposure for Pediatrics and Family Medicine, and to a certain extent, even for Surgery is becoming difficult. In the future coming days, the Pediatrics rotation will completely dependent on the outpatient clinic. I hope this kind of study can be replicated in the USA too. One comment which I would like to mention which is already mentioned by previous reviewers is, reaching saturation based on one interview is not a common practice in qualitative analysis. And also another comment is NVivo can only do transcribing but can't help with coding and themes. Overall this paper is very well written and can be useful for clinical academic administrators and curriculum developers.

Reviewer Expertise:

NA

No decision status is available

# Reviewer report: Reviewer response for version 1

**DOI:** 10.21956/mep.19810.r29826

**Published:** 2019-04-13

**Authors:** Felix Silwimba

**Affiliations:** 1 University of Lusaka

This review has been migrated. The reviewer awarded 5 stars out of 5 I have enjoyed reading through this study. it is a good study and brings out significant findings that directly speak to medical education in context. I agree the response rates were low but the the findings and conclusions are relevant. I recommend such a study to be replicated in low and middle income countries. Medical education is closely linked to physician competencies.

Reviewer Expertise:

NA

No decision status is available

# Reviewer report: Reviewer response for version 1

**DOI:** 10.21956/mep.19810.r29823

**Published:** 2019-04-13

**Authors:** Balakrishnan Nair

**Affiliations:** 1 Centre for Medical Professional Development and University of Newcastle

This review has been migrated. The reviewer awarded 3 stars out of 5 Ambulatory care teaching is under -utilised, even though most patients are seen in this setting . So this study is worthwhile The authors concluded what we already know about adult learning; that it should be inter-active , learner centered, pitched at the right level with immediate feedback . They also found that the teachers enjoyed it but had the usual impediments like lack of time , space and not knowing where the students were at that stage So this is a good research project re-emaphasing some very good points . However the student response rate to the online survey was only 6% while the consultant response was above 10 % ; only 4 teachers were interviewed with 3 analysed. So I am not sure whether the conclusions reached saturation point

Reviewer Expertise:

NA

No decision status is available

# Reviewer report: Reviewer response for version 1

**DOI:** 10.21956/mep.19810.r29825

**Published:** 2019-04-12

**Authors:** Prerna Agarwal

**Affiliations:** 1 Datta Meghe Institute of Medical Sciences

This review has been migrated. The reviewer awarded 4 stars out of 5 It's a nice practical study portraying the needs of students as well as the methods of the teachers in clinical settings. The authors have critically analysed their results. The study can contribute to policies for reframing of teaching schedules and methods to be more learner centric and effective.

Reviewer Expertise:

NA

No decision status is available

# Reviewer report: Reviewer response for version 1

**DOI:** 10.21956/mep.19810.r29827

**Published:** 2019-04-12

**Authors:** Subha Ramani

**Affiliations:** 1 Harvard Medical School, Brigham and Women's Hospital

This review has been migrated. The reviewer awarded 4 stars out of 5 The authors have conducted a rather neat mixed methods study to explore perspectives of students and clinical supervisors regarding challenges of teaching in the outpatient setting and teaching styles that are effective. It is appropriate that the balance of inpatient and outpatient teaching for students has been shifting towards increasing outpatient teaching with short inpatient stays. As an outpatient clinical supervisor myself, I completely agree about the lack of time for dedicated teaching and lack of teaching space.The abstract is very clearly written and conveys the summary of this study. Mixed methodology is an appropriate study design to answer their study questions. They had good numbers of teachers and learners for the quantitative part of the study. I would comment that one cannot reach data saturation in a one-hour single focus group discussion. Data saturation is more applicable to grounded theory studies and multiple interviews or focus group discussions are needed with constant comparison of themes until no new themes are discovered. This is an inaccurate use of the term.One other comment is that investigators obtain themes and NVivo organizes them. The software does not perform the analysis. Also, codes and themes are different levels of qualitative analysis, data are usually not coded into themes.The discussion does highlight important aspects of outpatient teaching and valuable suggestions.The bibliography was quite adequate.Overall, well worth doing this study, methodology was good, writing was good and there are important messages for outpatient supervisors.My major feedback is that authors should review qualitative research some more and use terminology that is appropriate. The themes are actually very illuminating, but the writing of the qualitative aspects of the study can be enriched.

Reviewer Expertise:

NA

No decision status is available
